# A spatial transcriptomic atlas of the host response to oropharyngeal candidiasis

**DOI:** 10.1128/mbio.00849-25

**Published:** 2025-06-30

**Authors:** Sunna Nabeela, Hayden McSwiggin, Rubens Daniel Miserani Magalhaes, Eliciane Cevolani Mattos, Mohammad Mannan, John T. Dillon, Ashley Barbarino, Eman G. Youssef, Shakti Singh, Wei Yan, Ashraf S. Ibrahim, Heather R. Conti, Priya Uppuluri

**Affiliations:** 1 The Lundquist Institute for Biomedical Innovation at Harbor-University of California Los Angeles (UCLA) Medical Center, Torrance, California, USA; 2 Department of Biological Sciences, University of Toledo 7923, Toledo, Ohio, USA; 3 Department of Medicine, David Geffen School of Medicine at UCLA, Los Angeles, California, USA; Universidade de Sao Paulo, Ribeirao Preto, Sao Paulo, Brazil

**Keywords:** OPC, *Candida albicans*, M2 macrophages, SPRR, spatial transcriptomics

## Abstract

Oropharyngeal candidiasis (OPC) caused by the fungus *Candida albicans* triggers a robust inflammatory response that rapidly eliminates the bulk of the fungal load in the oral mucosa. While pro-inflammatory responses to OPC have been extensively studied, little is known about the counterbalance of immunity in the infection milieu that mitigates inflammatory damage. We employed 10× Visium Spatial Transcriptomics, a next-generation technology that preserves the spatial integrity of infected tongue tissues, enabling high-resolution mapping of the host microenvironment during *Candida* infection in a murine OPC model. This approach provided a view of cellular interactions and immune dynamics, revealing intricate crosstalk between distinct immune cell populations. Our findings highlight a previously underappreciated role of the type-2 immune response and M2 macrophages in maintaining tissue homeostasis, and the predicted role of platelet degranulation in facilitating disease resolution. Finally, we identified a novel family of small proline-rich antimicrobial proteins with potent antifungal activity. These molecules emerge as promising therapeutic candidates, offering a new avenue for combating OPC.

**IMPORTANCE:**

Oropharyngeal candidiasis (OPC), a fungal infection caused by *Candida albicans*, affects individuals with weakened immune systems. Our study used spatial transcriptomics, a cutting-edge technology that preserves tissue architecture while mapping immune interactions at high resolution. This approach allowed us to uncover previously unrecognized cellular crosstalk and regulatory pathways that shape the host response to OPC. We discovered that platelets, beyond their role in clotting, play a key role in antifungal defense. Additionally, M2 macrophages are important for resistance to OPC. Most notably, we identified a new family of antimicrobial proteins with strong antifungal properties, presenting promising therapeutic potential. By uncovering these overlooked immune mechanisms, our research findings may lead to better treatments for OPC, particularly in immunocompromised individuals.

## INTRODUCTION

Oropharyngeal candidiasis (OPC) is an opportunistic infection of the oral and esophageal mucosa caused by the fungus *Candida albicans*. The infection occurs in otherwise healthy individuals with impaired immunity, such as in newborns or the elderly, and predominantly afflicts immunocompromised patients, including those suffering from HIV/AIDS, those treated with immunosuppressants, and individuals on antibiotics (1, 2). Although not life-threatening, OPC can cause considerable morbidity and increased incidence of esophageal cancer (3, 4). The host response to OPC has been extensively studied in models of immunocompromised and knockout mice, providing robust and replicable immune outcomes aligning with human oral candidiasis studies (5–8).

During OPC, healthy mice generate a rapid and intense inflammatory response (6, 9). The first line of defense against OPC is the innate immune system (10). Pattern recognition receptors on the surface of macrophages and dendritic cells recognize β-glucans, mannans, or chitin on the surface of *Candida*. Activated innate cells phagocytose *Candida* and release pro-inflammatory cytokines (IL-1, TNF-α, IL-6) that help recruit more immune cells to the site of infection (1, 9). Some of these cytokines drive CD4+ T cells toward a T helper 17 (Th-17) phenotype (6). Th-17 differentiation is triggered by IL-6 and IL-23 and produces IL-17A, IL-17F, and IL-22 cytokines that further recruit innate immune cells to the site of infection (9). IL-17A and IL-17F also stimulate the production of antimicrobial peptides (AMPs) such as β-defensin 3 and S100A8/A9 from epithelial cells that help restrict *Candida* growth (9, 11). IL-22 further facilitates anti-inflammatory epithelial cell proliferation and repair (9). Overall, the invasion of oral epithelial cells by *C. albicans*, activation of innate immune cells such as neutrophils by pro-inflammatory cytokines and chemokines, and release of AMPs resolve OPC but can cause considerable inflammation and tissue damage (12).

A balance of the type 1 and type 2 signals is critical for long-term control of infection while also preventing extensive tissue damage by an exuberant inflammatory response. Ironically, little is known about the anti-inflammatory response during mucosal or disseminated candidiasis. The primary type-2 cytokines IL-4, IL-10, IL-13, and TGF-β polarize T-cells to the Th-2 phenotype and induce alternative M2 macrophages and pro-repair cytokines (13, 14). Furthermore, “pleiotropic” immunoregulatory cytokines such as IL-6, IL-10, and TGF-β have only been investigated for their pro-inflammatory properties during candidiasis (9, 15, 16). While the protective inflammatory host response that clears the fungi has been well studied over the past two decades, hardly anything is known about the sequence of events that occur in parallel that help equilibrate inflammation and initiate tissue repair while the fungal infection is being eradicated.

We used spatial transcriptomics (Visium 10×) to characterize the comprehensive transcriptional patterning and regulation of host response genes in the infected tissue during OPC in immunocompetent mice. This unique technology obviates the need for tissue dissociation and preserves the spatial context of cells. We used fresh frozen mouse tongues infected for 2 days, a time point that harbored a significant fungal burden while repair mechanisms began. An outstanding feature of spatial transcriptomics is that it combines high-definition tissue imaging with unbiased spatially defined RNA sequencing (RNA-seq) using barcoded spots (55 µm) on glass slides (17). Transcriptomic changes are observed in the tongue mucosa using high-resolution *in situ* sequencing. Thus, one can visually map host genes overrepresented in the exact area of the pathogen gene expression or disease and compare it to other sections of the same tissue that are not infected or have already recovered from infection.

Our findings shed light on the determinants of a strong type 2 response that arises after the Th1 response subsides at day 2. The infected tissue consisted of an increased presence of alternative type 2 macrophages rather than inflammatory M1 macrophages, neutrophil degranulation, megakaryocyte expansion, cytokine markers, and platelets for wound repair and infiltration of antimicrobial peptides to combat infection, as well as to simultaneously initiate wound healing. Comparison of infected loci versus infection-resolved sections on the same tissue also sheds light on the significant changes in metabolic reprogramming and alterations in extracellular matrix reorganization between inflamed cells and the tongue cells healing from infection. Additionally, our results unravel a novel and large family of small proline-rich antimicrobial proteins (*SPRR*) that are expressed at the highest levels in the infected tissue. Recombinantly produced *SPRR* displayed direct *Candida* killing by damaging fungal plasma membranes *in vitro*. Furthermore*,* an oral treatment with *SPRR* significantly reduced fungal burdens in the tongues of steroid-treated mice during OPC. Finally, double knockout mice that are deficient in both *SPRR1* and *SPRR2* were significantly more susceptible to OPC than wild-type immunocompetent mice, indicating that these antimicrobial proteins are likely important for resistance to OPC. Overall, our studies visually highlight the transcriptional landscape of advanced stages of OPC and provide several hypotheses driving ideas that could help to mechanistically explore the type 2 host immune response and wound repair processes prevalent during OPC.

## RESULTS

### Spatial transcriptomics of resolving OPC identifies four major tissue compartments in uninfected and infected tongue

**Fig 1:**
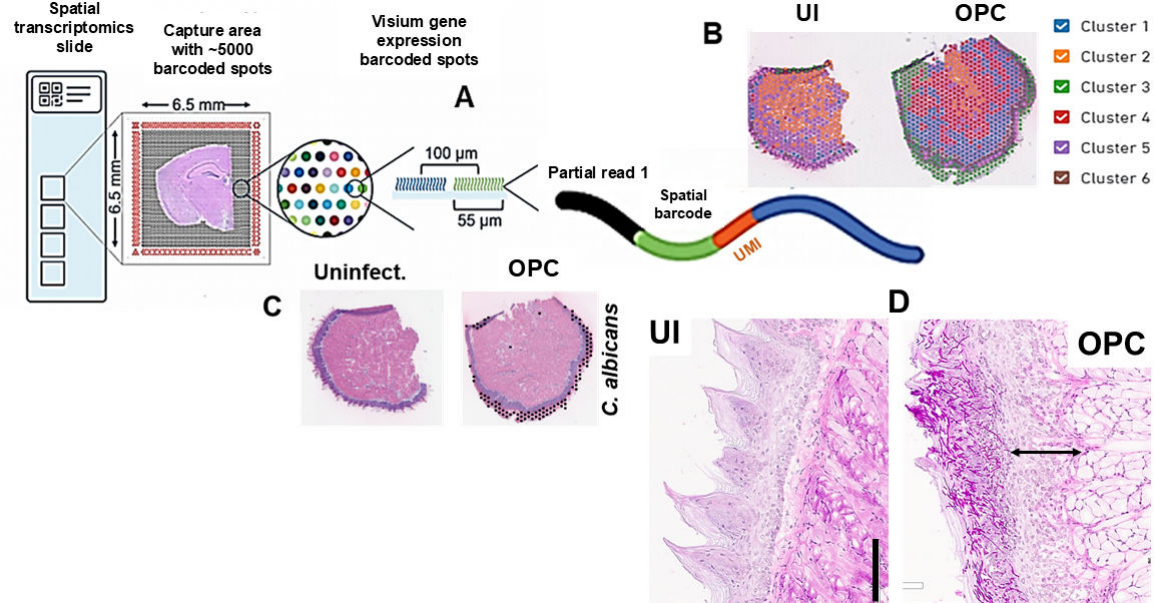
Overview of steps in 10× spatial genomics. (**A**) After the tissue is fixed and cryosectioned to a 10 µm thickness, it is placed on a slide that contains ~5,000 capture probes, each. Sections are hematoxylin and eosin (H&E) stained and imaged, then permeabilized to release RNA, which binds to adjacent capture probes (barcoded mRNA binding nucleotides), allowing for the capture of gene expression. Barcoded cDNA is synthesized from captured RNA, and sequencing libraries are prepared. Standard short-read sequencing of 10× barcoded libraries = transcription profiling of 1,000’s of individual locations. (**B**) Bound genetic data is “GPS-mapped” to its original spot and visualized spatially. Data is presented in clusters and annotated to different cell types based on expression. E.g., Cluster 3 green represents *C. albicans* gene expression expressed exclusively in infected tissues. (**C**) H&E-stained uninfected (UI) and OPC-infected tissues with the presence of *C. albicans* identified by black dots. (**D**) Periodic Acid-Schiff (PAS)-stained UI and OPC tissues display a healthy epithelial architecture in UI, while damaged squamous epithelial cells, destroyed lingual papillae, and a thick lamina propria in OPC. Scale bar = 200 µM.

To analyze the microenvironment during OPC, we employed the 10× Genomics Visium spatial transcriptomics technology on frozen tissue sections (*n* = 4) from tongues of normal and *C. albicans*-infected Balb/c mice at 60 h of OPC (hereby referred to as day 2, for ease of representation). At this time point, there was a significant existence of infection that could be visualized by Periodic Acid-Schiff staining (PAS) staining of the tongue sections (Fig. 1).

While uninfected (UI) tissues displayed a healthy architecture of the epithelial tissue, signified by lingual papillae and a thin lamina propria, infected sections harbored abundant *C. albicans* hyphae within the squamous epithelial cells, destroyed lingual papillae, and a thick lamina propria. Visium slides permit concurrent viewing of hematoxylin and eosin (H&E)-stained histology sections as well as 55 micron diameter capture spots that detect local mRNAs (Fig. 1). These individually barcoded capture spots allow identification of putative cell types and differentially regulated genes (DEG) based on mRNA expression within each spot across the tissue section. Combined analyses of the four respective tissue replicates revealed a significantly high correlation of expression patterns between replicates, as demonstrated by the Uniform Manifold Approximation and Projection for Dimension Reduction (UMAP) projections and Principal Component Analysis (PCA) plots (Fig. S1).

**Fig 2:**
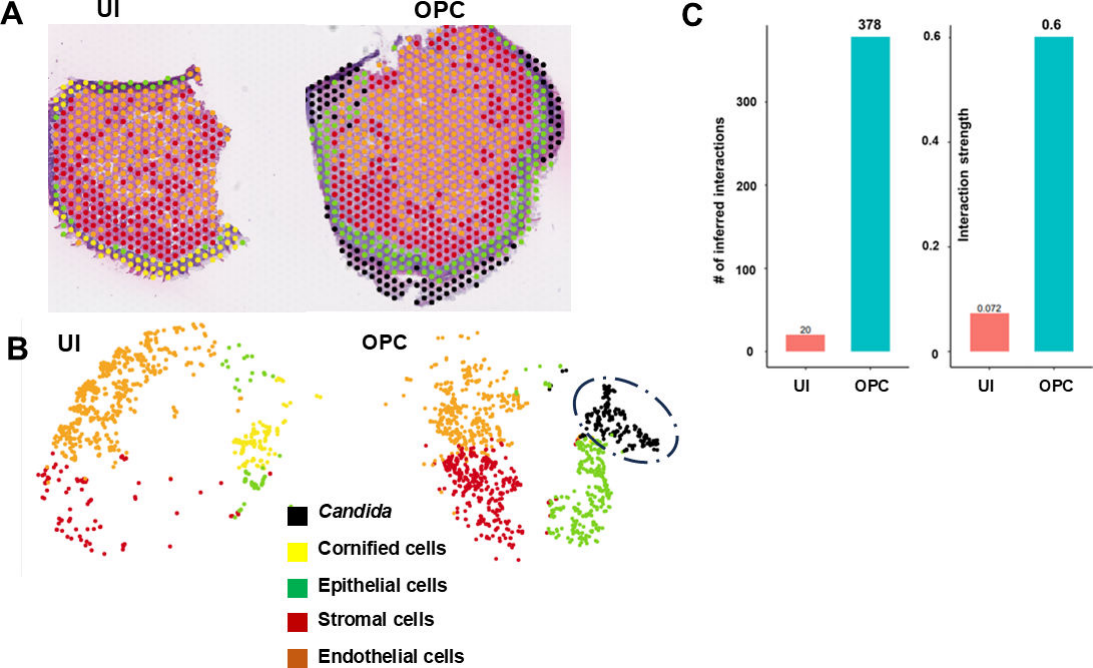
Major cell type annotation. Spatially resolved transcriptome (**A**) and t-SNE plots (**B**) showing representation of five major cell types in the tongue tissue. (**C**) Cell Chat software revealed the number and strength of inferred cellular signaling interactions from the spatial transcriptomics data.

Dimensionality reduction was performed using UMAP visualization to cluster ~7,270 individual capture spots from all eight tongue samples. Spatial projections of the data set yielded gene expression data from 23,625 genes (18,641 host genes and 4,984 *C*. *albicans* genes). Using the transcriptomic signatures of key gene biomarkers and utilizing the Seurat batch correction software combined with ShinyGo 8.0, we identified four major cell compartments in the tongue tissues: cornified envelope cells (Lor, Sprr), epithelial cells (Krt13, Krt14, Krt5), fibroblasts/stromal cells (Col1a1, Col3a1, Lum), and endothelial cells (Vwf, Pecam1, Cldn5) (Fig. 2A). Additionally, *C. albicans* gene expression (ADH1, ECE1) was used to identify the infection loci sections. As expected, uninfected tissues did not show fungal mRNA. The cornified layer (yellow) was overrun by *C. albicans* infection (black) in infected tissues, and the UMAP of the clusters showed that *Candida* cells clustered closely with squamous epithelial cells (green; Fig. 2B, also Fig. S1). The gene lists associated with each of the cell types are included in Table S1.

**Fig 3:**
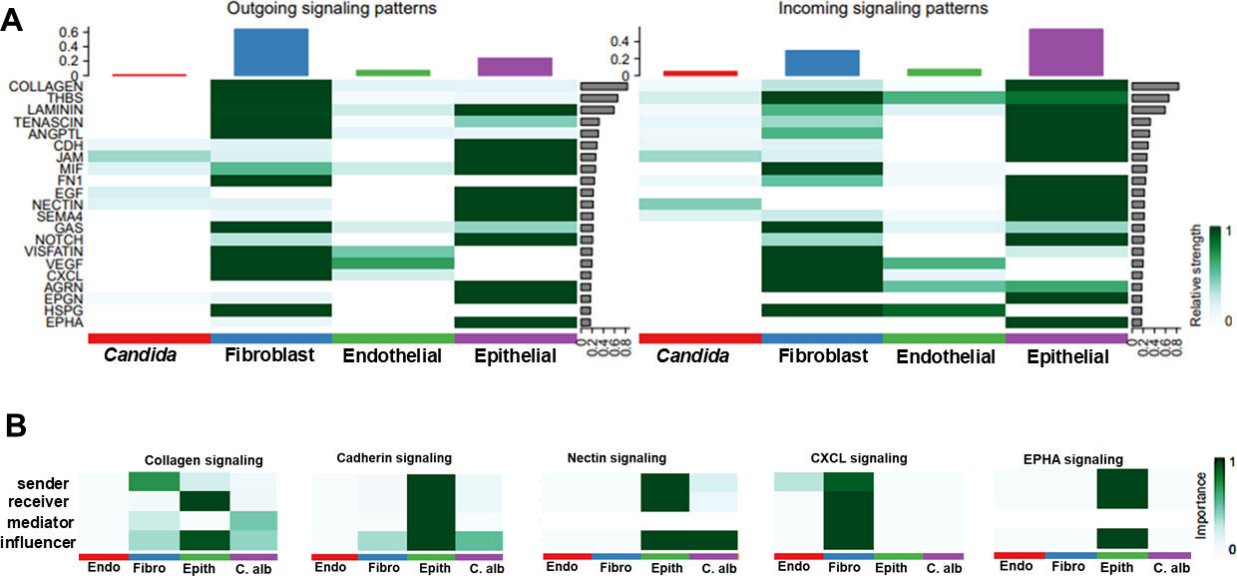
Cell chat identified signaling direction and genes. (**A**) Cell chat demonstrated outgoing and incoming signaling patterns, signifying communication between the four major cell types. (**B**) The communication was further elaborated on the cell type playing the influencer or mediator of the communication between the other major cell types and the processes active during infection

We applied CellChat (18), the latest cell-cell communication analytic algorithm, to our data set. CellChat builds a model where various cell types annotated from the spatial data are represented as nodes and their interactions as edges. If a node (i.e., cell type such as fibroblast or *Candida*) is highly interactive and central to the communication network, then it is identified as an influencer. We first found that the overall communication number was enhanced in infected cells compared with controls, with ~378 signaling pathways communicating between different cell types with high interaction strength (Fig. 2C). Most intercellular communication with high strength during infection occurred from fibroblast to epithelial cells via pathways involved in cell-cell adhesion/proliferation (Nectin, Cdh, Jaml), extracellular matrix remodeling (Collagen, laminin), growth factor action (Egf, Vrgf, Epgn), or immunomodulation (Cxcl, Mif, Sema4) (Fig. 3A). Furthermore, we measured the capability of cell types as gatekeepers to mediate or influence communication flow between two cell groups. As examples, we found that while collagen signaling occurred from fibroblasts (sender) to basal/suprabasal epithelium (receiver), it was influenced by the cell types associated with *C. albicans* (present in the cornified epithelium). Similarly, E and P-cadherin (CDH) or nectin signaling was contained within the epithelium but influenced by the infected cells. On the other hand, *CXCL* signaling or *EPHA* signaling primarily was mediated within the fibroblast or epithelial cells, respectively (Fig. 3B). Other significantly represented elements with various other directional signaling patterns are shown in Fig. S2. Overall, the results showed that determinants regulating the organization and proliferation of extracellular matrix compartments and cellular remodeling are abundant during OPC.

### Differential gene expression revealed a balanced immune response during OPC

Differential gene expression analyses (DEG) between OPC and non-infected tissues were carried out using Seurat to identify the most significantly upregulated genes by *P*-value and L2FC (Log2 fold change) values. A total of ~1,850 genes were upregulated, and 850 genes downregulated in infected versus the control tissues, *P* < 0.05 and Log2 > 0.8 (>1.75-fold) (Table S2).

**Fig 4:**
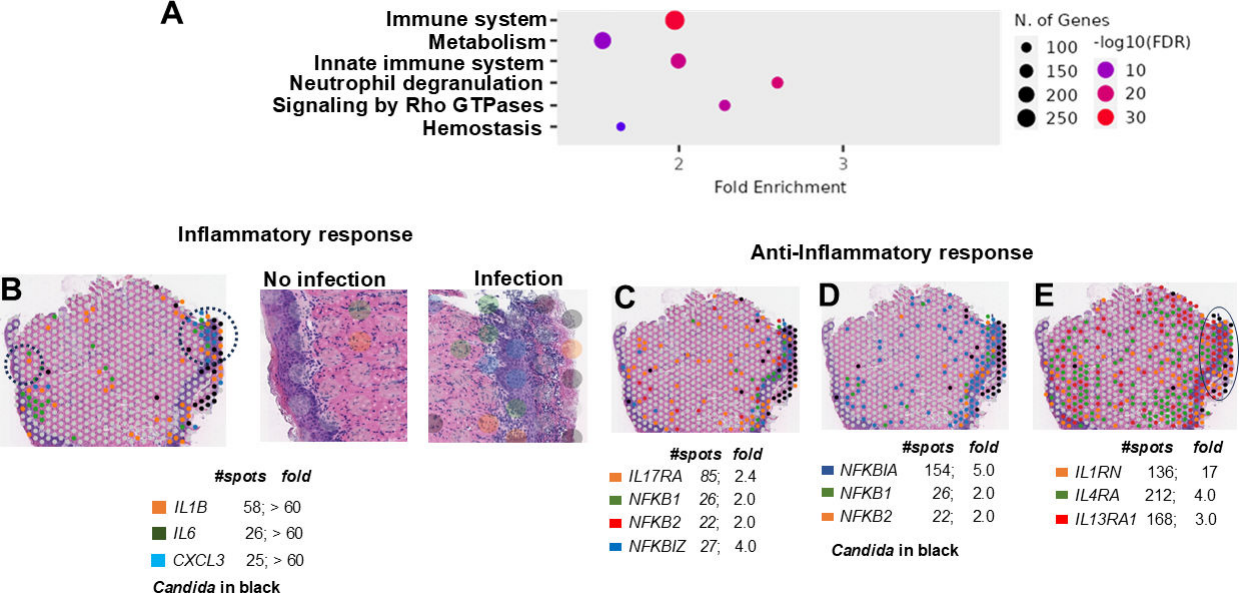
Predominant GO terms significantly upregulated in OPC versus UI and spatial representation of differentially expressed and positioned inflammatory versus anti-inflammatory genes. (**A**) The top six significantly elevated processes in OPC versus UI were identified based on DEG and using ShinyGo, followed by KEGG or Reactome. (**B**) Inflammatory cytokines were concentrated exactly in the milieu of fungal cells and expressed at high levels; these were not expressed in the area without *C. albicans* (black dots). (**C**) NF-κB family transcription factors are also upregulated in the infection milieu throughout the epithelial region, albeit at a lower strength. (**D**) NFKB inhibitors showed a robust presence in the same milieu as NFKB and expressed at larger levels than NF-KB itself. (**E**) Type 2-associated markers were spatially distributed at a significantly larger scale than inflammatory genes. UI tissues did not express these genes at significant levels and hence are not shown here for brevity. *C. albicans* are shown as black dots in **(B)** to **(E).**

ShinyGo and two other pathway recognition databases, such as Reactome and Kyoto Encyclopedia of Genes and Genomes (KEGG) (19–21), unraveled six predominant Gene Ontology (GO) terms representing the upregulated genes. These encompassed the immune system as the most regulated function (False Discovery Rate [FDR] 1 × 10^−20^; over 17% of all upregulated genes), with a majority of these signifying the innate immune system and neutrophil degranulation (Fig. 4A). Within the innate immune system, cytokine-cytokine receptor interaction was one of the topmost enriched GO terms, and unsurprisingly, the infected tissues also displayed a significant presence of the IL-17 signaling and NF-KB signaling pathways (Table S2). The second most elevated process was metabolic pathways that encompassed the overrepresentation of nucleotide metabolism, glycolysis, and glutathione metabolism (with almost all primary enzymes of the latter process, e.g., *GPX1-3, GSTA1-4* upregulated) (Table S2). These pathways are important for antioxidant activities and reinforcing cellular homeostasis (22). Signaling by Rho GTPases that specifically activate formins and regulate actin cytoskeletal dynamics and cell migration (23) was also elevated (115 genes elevated >2-fold), followed by 86 genes categorized in the GO term “hemostasis,” indicating the early phase of wound repair (24) (Table S2). When this GO term was further sorted for function, the topmost enriched genes were those involved in platelet activation, signaling, and aggregation, making up 50% of the hemostasis genes. Also included were related factors involved in megakaryocyte development and platelet differentiation that indicated an early phase of wound healing (Pf4, Selp). Furthermore, other genes in this category also have roles in cell surface interactions with the vascular endothelia, such as integrins (*Itgb2, Itgb3*) (25)*, Cd44* (26)*, Jaml* (27)*, Cd177* (28), and *Ceacam1* (29).

The connection to hemostasis and platelet function was further supported by the robust presence of over 40 genes involved in platelet activation, signaling, and degranulation in the infection milieu (Table S2). This included genes such as (*Srgn, Selp, Thbs1, Orm2*), which were also elevated >3-fold when infected tissues were validated for expression by quantitative PCR (qPCR) (Fig. S3B). Oral megakaryocytes localize to the tongue tissue and are involved in protection against OPC in C57Bl/6 mice (30). The CD41^+^CD11b^−^Gr-1^−^F4/80^−^ megakaryocyte population also expanded in the tongue tissue of BALB/c mice during infection, starting 2 days after *Candida* exposure, with continued expansion by day 4 (Fig. S3C).

The limitation of Visium spatial transcriptomics is its insufficient resolution to separate single-cell transcriptomic profiles. This is because each spot captures several cells and cell types within the tissues, resulting in gene expression with varying degrees of heterogeneity. This overlap makes it challenging to differentiate and categorize distinct cell types. The data were further analyzed by comparing the distribution of gene expression across the tissue spots with the expression levels of those genes. This was done because certain genes, despite being expressed at lower levels, were found across many spots, covering a larger surface area. In contrast, other genes showed high expression levels but were concentrated in fewer spots. This latter scenario was true for the pro-inflammatory cytokines such as *IL-6*, *Cxcl3,* or *IL1b*, that were >60-fold highly expressed in infected tissue macrophages but occupied a cumulative of only ~110 spots in the whole tissue (Fig. 4B). The expression was the highest only in regions of the tissue that had not resolved *C. albicans*. On the other hand, parts of the tissue with resolved infection did not display expression of pro-inflammatory cytokines such as *Cxcl3* (Fig. 4B).

Probing further, the OPC tissue sections displayed components of both inflammatory and anti-inflammatory reactions. Protective IL-17 signaling pathway (*IL-17ra, IL-17a, IL17F, IL-22, IL-23a*) and *NF_K_B* (*Nfkb1* and *Nfkb2*), including the newly identified IL-17-dependent non-canonical NF_k_B transcription factor Ikbzeta, *Nfkbiz* (8) were visualized in close proximity to the infected epithelial cells (Fig. 4C). Also, genes categorized as being a part of the *NF-kB* inhibitory pathway, which play a crucial role in regulating the production of Th-17 cytokines, *Nfkbia, Nfkbie, Nfkbid,* were expressed twofold higher than *NfkB* at the same infection loci (Table S2). *Nfkbia* alone was much more widespread as it was found in 154 spots versus only 48 spots that harbored *Nfkb1* and *Nfkb2* (Fig. 4D). This indicated a milieu with diminishing inflammation.

Indeed, anti-inflammatory genes exhibited a considerable presence in the infection setting; the IL-10 family of genes important for wound healing and repair (*IL1r2, IL1r1, IL-24, IL-19, IL-22*) were elevated >2- to 60-fold, although *IL-10* itself remained unchanged. Importantly, there was the presence of other anti-inflammatory markers with larger distribution in the infected tissue. These were the IL-1 antagonist *IL1rn* (31)*,* and the type 2 cytokine receptors *IL-4ra* and *IL-13ra1* (32) (*P* value 1 × 10^−16^; FDR 1 × 10^−14^). These three genes alone spanned ~520 spots (five times more than the inflammatory response) and were expressed by keratinocytes in the inflamed locus of infection that also harbored genes of several pro-inflammatory cytokines (Fig. 4E). Interestingly, genes encoding for Th-2 cytokines *IL-4* or *IL-13* that trigger expression of these receptors (32) were themselves not detected. Data associated with each of these pathways are found in Table S2; Fig. 4B through D shows the number and distribution of spots vis-à-vis the location and gene expression levels of the host response genes. The control uninfected tissues showed significantly low basal expression levels of the gene markers as displayed in Fig. S3A. Overall, the results showed that the infection milieu was dominated by both pro- and anti-inflammatory immune response genes, with the latter being at least three times more predominant, in the late stages of OPC.

The expression levels of some genes were independently verified in four biological replicates of infection, by quantitative reverse transcription PCR (qRT-PCR), and were consistent with the expression patterns observed in the spatial study on infected tissues (Fig. S3B). The qPCR further highlighted that inflammatory cytokines such as *IL-1β* or *IL-17a* are expressed at the highest levels during early infection at day 1, as expected. While their levels reduced in comparison at day 2, they were still >10-fold elevated compared to uninfected tissues, validating the spatial data. Both in the qPCR and the spatial outcomes, *IL-1β* had a multifold abundance over *IL-1a* (10-fold vs 1.5-fold expression during infection). The absence of Th-1 cytokines was corroborated by the complete absence of *IFN-γ* and low levels of *TNFα* in the infection milieu.

### OPC tissues showed a robust presence of myeloid cells

Cell-type enrichment analysis using DESCARTES = DEvelopmental Single Cell Atlas of gene RegulaTion and ExpreSion; descartes.brotmanbaty.org (Descartes Cell Types) identified an immune signature associated predominantly with monocytes or macrophages over neutrophils (Table S3). This was also seen in qPCR, where neutrophil markers such as Cd11b/Itgam were expressed at high levels on day 1 but completely disappeared by day 2 (Fig. S3B). Neutrophil analyses by flow cytometry also displayed a statistically significant reduction in their numbers at day 2 (Fig. S3D) compared to day 1 of OPC. However, there was a striking predominance of neutrophil degranulation genes (Fig. 4A; Table S2). Over 125 myeloid-associated genes were elevated during OPC, and one-third of these were further categorized as the anti-inflammatory M2 macrophages (Table S3).

**Fig 5:**
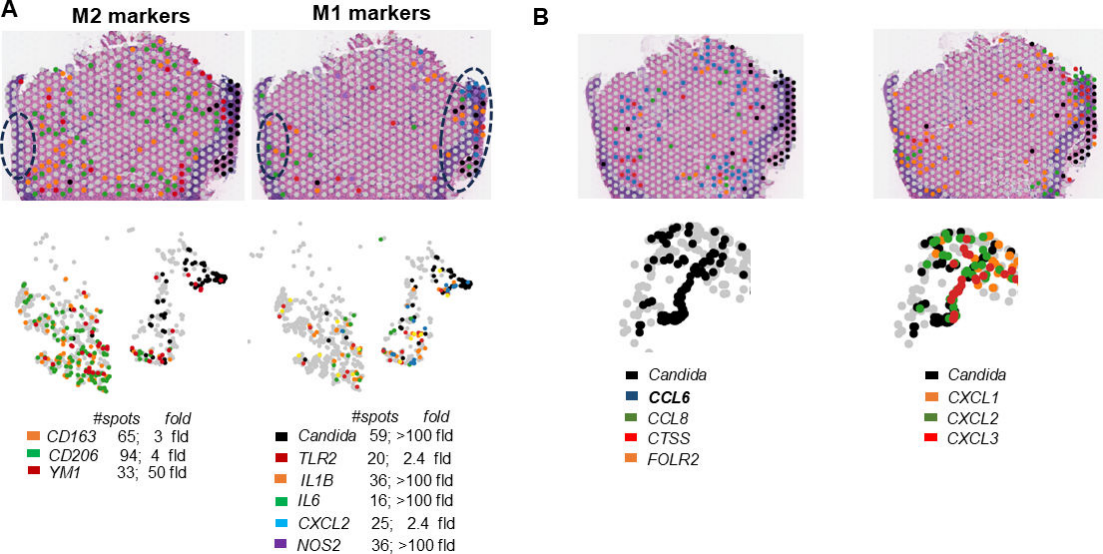
Spatial representation of differentially expressed and positioned M1 versus M2 macrophage genes. (**A**) Correlates of M2 and M1 macrophage markers were identified in the OPC tongue tissue. Top panel is the spatial representation of the location of these macrophages, and the bottom panel is the corresponding t-SNE plots of the same tissues. Numbers enumerate spots of each gene and fold change of each gene compared to UI tissue. (**B**) Identifies M2 (left) associated genes by pulling out the features of CCL6, also showing that these genes are not expressed alongside *C. albicans,* portrayed in black. Right panel identifies M1-associated chemokines, also indicating that these genes are expressed in the infected locus.

Given the presence of anti-inflammatory markers in our spatial data set, we questioned if M2 macrophages that function in dampening inflammation and wound healing indeed had a significant presence over the classical pro-inflammatory M1 macrophage markers. Signature M2 macrophage markers such as the macrophage mannose receptor *MRC1/YM1, CD163,* and *CHIL3/CD206* were >3 times in abundance in the tissue compared to the M1 macrophage cell type as observed by overall spot distribution numbers and average % expression of individual genes representing each M1 or M2 cell type (for example dot plot in Fig. S4A and spatial distribution in Fig. 5A). Furthermore, when a predominant M2 macrophage polarizing cytokine *CCL6* was pulled out, several macrophage genes such as *Cd68, Ccl9, C1qa, C1qb, C1qc, Clec10a* clustered along with it (Table S4). Importantly, several genes fundamentally associated with the M2 macrophage cell type, such as *Ccl8* (a M2 chemoattractant), *Folr2,* and *Mrc1* (receptor biomarkers of M2), besides several other M2 marker genes, also specifically clustered with *Ccl6* (S4 and Fig. 5B). No other genes other than the macrophage (and M2) cell types clustered along with *Ccl6,* and extracting features from *Mrc1* also in turn identified *Ccl6*. This indicated that the environment exclusively portrayed an M2 milieu. The uninfected tissues had a negligible presence of macrophage cells. Finally, qRT-PCR verification of two key M2 markers, *Mrc1* and *Arg1,* revealed that the M2 markers were up-expressed beyond day 2 (Fig. S3B).

Similarly, since IL-1β is an immunomodulator, we inquired if it clustered with pro- or anti-inflammatory cell types. We found that *IL-1β* clustered along with the macrophage marker *Cd14* and other biomarkers of M1 macrophages, such as *Cd80, Tlr2, Nos2, Fpr1,* and *Csf3r*. This cell type/environment also displayed >2-fold expression of at least 37 genes associated with the IL-17 signaling pathway, including those of the NF-KB signaling pathway, and >30-fold expression of pro-inflammatory chemokines *Cxcl1, Cxcl2,* and *Cxcl3* (Fig. 5B; Table S4). Furthermore, as seen previously with respect to the presence of pro-inflammatory cytokines specifically in the loci of infection, this data reinforced that the inflammatory M1 macrophage markers were also largely concentrated within the locus that had *Candida* presence, and cell type annotations revealed that M1 macrophages were the source of the inflammatory cytokines. On the other hand, M2 macrophages were localized throughout the tissue (Fig. 5B) and found largely excluded from the direct area of fungal infection.

**Fig 6:**
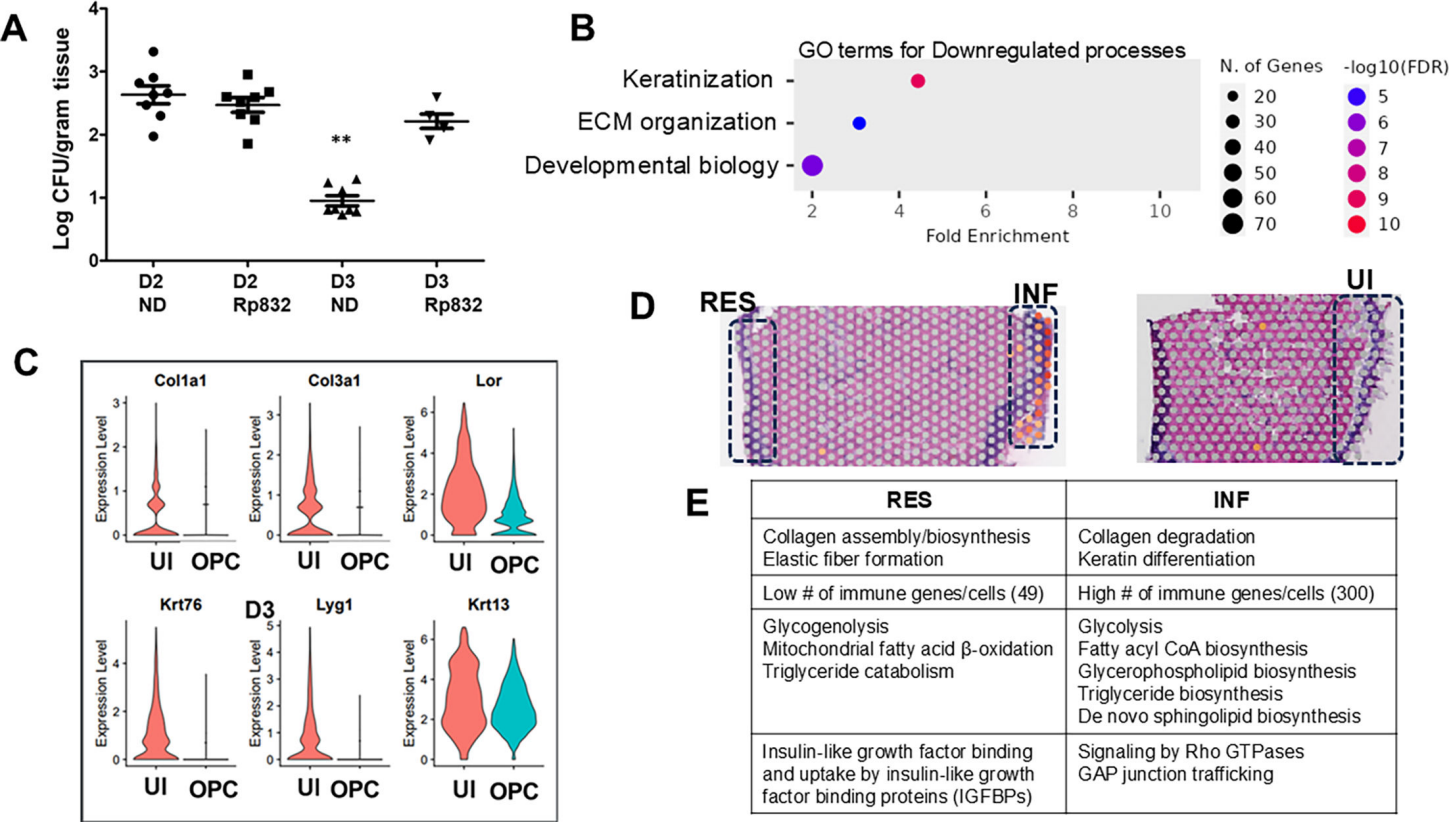
Predominant GO terms and genes significantly downregulated in OPC versus UI; metabolic pathways differentially regulated in OPC infected (INF) versus resolved (RES) milieu. (**A**) Immunocompetent mice (*n* = 8) were intraperitoneally treated with 125 µg of the M2 macrophage inhibitor Rp832 1 day prior to infection and 1 day following infection. Tongues of drug-treated and untreated mice were harvested at day 2 (D2) and D3 post-infection, and CFUs plotted in GraphPad Prism 5. **P < 0.01 versus all other conditions. Dot plot identifying the top 3 most enriched GO terms downregulated in OPC versus UI. (**B**) Violin plots of cornified epithelial and structural keratin genes downregulated in OPC versus UI. (**C**) Identification of the INF milieu, which shows colored spots that indicate *Candida* presence (*C. albicans* ADH1 gene marker). The RES part does not harbor *Candida* but shows that the squamous epithelial layer is still not as healthy as UI, which has vigorous lingual papillae. (**D**) Metabolic pathways upregulated in RES versus INF milieu in the same OPC tissue. (E) Summary of the differences in metabolic status between RES and INF sections in the same tissue.

To understand if M2 macrophages have a role to play in resistance to OPC, we used the peptide RP-832C that targets Cd206 (Mrc1) receptor on M2 macrophages and reduces their activity (33). Immunocompetent mice were treated with RP-832C on day −1, day 0, day 1, and day 2 with respect to infection, and fungal burden was measured at days 2 and 3. Depletion of the M2 population in the tongue was confirmed by qRT-PCR of M2 markers *Mrc1*, *Arg1,* and *Cd163*. Results exhibited that at day 2, both placebo and inhibitor-treated mice had a similar average log fungal burden of 2.5. However, at day 3, while the placebo-treated mice saw a complete clearance of infection as expected, M2 inhibition prolonged the persistence of infection, with fungal burdens maintained at 2.2 log (Fig. 6A). This indicated that M2 macrophages may have a role to play in OPC clearance.

### Infected tissues display keratinocyte differentiation indicative of an inflammatory response during wound healing

Three biological categories stood out as significantly downregulated in infected tissues: keratinization, extracellular matrix organization, and developmental biology (Fig. 6B; Table S5). Infected tissues exhibited a striking decrease in cornified envelope genes, likely due to the destruction of the cornified epithelium by infection.

Importantly, 42 out of a total of 54 keratin (KRT) genes largely involved in maintaining the structural integrity of epithelial cells were downregulated twofold to sevenfold during OPC. These included the markers specific for suprabasal oral keratinocytes *Krt4* and *Krt13* (34), which were decreased threefold to fivefold. Interestingly, the only *Krt* genes upregulated were 11 wound-activated keratinocyte genes also important for immunomodulation (35), including *Krt6a, Krt6b, Krt16,* and *Krt17*. Indeed, the infection milieu in the epithelium had the immune system as the predominant GO term, and these *Krt* genes are known to also promote a Th-1/Th-17-dominated immune environment (36). Remarkably, while these genes were 3–10-fold up in infected tissues, they were also highly active in the baseline unwounded/uninfected tongue epithelium, indicating that the tongue epithelium is primed for wound repair (Fig. S4B). Furthermore, the uninfected tongues also displayed high baseline levels of Annexin-A1 (*Anxa1*) and *Slpi*, two genes encoding proteins that ameliorate wound healing by counteracting inflammation (37, 38). In infected tissues, the expression of these genes increased threefold to eightfold, pointing to their importance in anti-inflammation and repair. Other genes linked to epithelial and immune cell migration, such as those that encode for S100 proteins, defensins, serpins, and annexins, were also upregulated in the infected but not in the uninfected tissue (Table S5).

Similarly, several genes encoding collagen 1, 3, 4, 5, and 6 were downregulated in the infected tissues (Fig. 6C), indicating a hindrance in collagen crosslinking functions and degradation of extracellular matrix. Correspondingly, several collagen degradation protein-encoding matrix metalloprotease genes, such as *Mmp3* and *Mmp8-9*, were upregulated >20- to 70-fold. Gene lists for up- and downregulated categories are presented in Table S2. Collagen degradation is an essential early step in subsequent extracellular matrix remodeling, and activation of Mmp9 plays an important role in the restoration of epithelial morphology after tissue damage (39). Overall, the day 2 environment was consistent with the early phase of wound repair with the presence of M1 and M2 macrophages, immunomodulatory keratins, and matrix metalloproteases active in the milieu (see heatmap Fig. S4), also including cytokines important for wound healing such as IL-22 and IL-24. When IL-24 features were analyzed, they revealed the likely role of this cytokine in leukocyte trans-endothelial migration, with VEGA pathway and ephrin signaling pathway genes enriched >2-fold, supporting the contribution of IL-24 in cell-cell communication (40, 41) (Table S6).

Taking the analysis a step further, we observed that in a set of biological replicates, one half of the tissue had resolved the infection (RES), while the other half was still infected (INF) (Fig. 6D). RES section of the tissue demonstrated destroyed lingual papillae and a thick lamina propria due to prior infection. We considered this an excellent opportunity to fully harness the advantages of spatial technology, to understand the two seemingly disparate environments in the same tissue. Furthermore, this narrowed comparison allowed us to dissect the RES milieu in a detailed way. Transcriptome outcomes placed RES as an intermediate stage between UI and INF environments. Compared to INF, RES displayed increased collagen biosynthesis with representation of genes involved in assembly of collagen fibrils, elastic fiber formation, and microautophagy (Table S7)—processes important for extracellular matrix (ECM) repair and those that were strikingly downregulated in INF when compared to UI. Additionally, in contrast to the INF environment that displayed matrix metalloproteases (*Mmp3, Mmp8, Mmp9, Mmp13*) with extensive capability of supervising the signaling of chemokines (42); RES instead exhibited upregulation of *Mmp2* and *Mmp19* that allow migration of fibroblasts during angiogenesis and dampening inflammation with positive effects on tissue repair (43, 44). Indeed, compared to UI, only 49 immune-associated genes were upregulated in RES versus over 300 genes in the INF milieu (Tables S2 and S7). RES showed the presence of antimicrobial proteins and cytokines such as *Cxcl12* that specifically influence wound healing through fibroblast-specific Vascular Endothelial Growth Factor (VEGF) expression (45). Thus, the RES milieu aligned with the proliferative phase of wound healing with reduction in inflammation, increased angiogenesis, and deposition of collagen.

One GO term that showed the most dominant changes in comparison between INF and RES was metabolism. INF displayed upregulation of cholesterol biosynthesis, glycolysis, and biosynthesis of fatty acyl CoA, glycerophospholipids, triglycerides biosynthesis, and *de novo* synthesis of sphingolipids. RES, on the other hand, exhibited glycogenolysis, mitochondrial fatty acid beta-oxidation, and triglyceride catabolism. It has been reported that upregulation of glycolytic metabolism is prevalent in the inflammatory phase of repair (46). Later, the proliferation or remodeling stages cause a switch toward oxidative metabolism (47) as seen in RES (Fig. 6E).

### *Candida* response

OPC is an infection of the immunosuppressed and is considered a risk factor for oral and esophageal cancer (5). An advantage of spatial transcriptomics is that it can simultaneously reveal gene expression patterns of both the host and the pathogen, which can be visualized and examined in perspective to each other. Considering that UI tissue did not have fungal cells, infected tissues were studied for the top 500 of the >4,000 *C*. *albicans* genes found expressed in the infection milieu (Table S2). *C. albicans ECE1*, a gene encoding candidalysin, was one of the top 3 upregulated genes, with *IDH1, ENO1, RBT5,* and *ACS1* making up the top 5 most expressed genes. The property of Eno1 as the foremost antigen in patients with candidiasis is well known (48). Also, it has been previously shown that GPI anchor proteins (Ihd1 and Rbt5) are important and overrepresented during OPC (49). Over 50 hyphal genes (including *HYR1, HWP1, ALS3*) were found upregulated >10-fold, while genes associated with the yeast phasic growth (*PES1*, *CLN3*, *YWP1, NRG1*) were not found in the top 500 highly expressed genes in the infected tissues (Table S2). This outcome fits well with the observation that hyphae are prominent in infected tissue samples (50). However, this may not indicate that the infectious milieu does not have yeast cells. It is likely that they are easier to be eliminated by the innate immune cells when produced, whereas hyphae are not. While almost all *C. albicans* genes found elevated during OPC from previous studies, such as by Fanning et al. (49), were also discovered in our data set, several novel genes encoding antigenic proteins were found. For example, the glyoxalase GLX3, the triose-phosphate isomerase TPI1, and glutathione peroxidase GPX3 with roles in resistance to oxidative stress (51); and genes involved in gluconeogenesis and tricarboxylic acid (TCA) cycle, indicating these metabolic pathways are important during OPC infection. The intention of this study was to focus on the host immune response. Fungal gene expression was only used to serve as markers for the infected loci. Further analyses of the fungal transcriptome will need to be done. The 10 most regulated processes and top 500 genes upregulated >15-fold during OPC are elucidated in Table S2 and can be used for future studies.

### Antimicrobial small proline-rich proteins are the highest-elevated genes in OPC

Compared to UI tissues, infected tongue sections exhibited an overwhelming breadth and intensity of gene expression by a family of small proline-rich proteins produced by the epithelial cells called SPRR (52). One of the proteins, *Sprr2e*, was found to cluster with all of the other 13 members of the *SPRR* family. The *SPRR*s were also found to be enriched with other genes coding for S100A8 (calgranulin A) and S100A9 (calgranulin B) (*P* < e^−50^), which form an antimicrobial heterodimeric complex also known as calprotectin in keratinocyte cells (53). Additionally, beta defensins (*Defb14, Defb1*) were also co-expressed along with the SPRRs with the same level of significance as calprotectin, making up the top 20 of genes co-regulated in the same locus as other anti-microbial proteins. Perhaps to sustain their presence and prevent degradation, this set of highly upregulated genes also included two cysteine-type endopeptidases and protease inhibitors *Csta1* and *Cstdc5* (Table S8). Since EGFR signaling stimulates epidermal proliferation and keratinization, it was also found to cluster with *SPRR* (Table S8).

**Fig 7:**
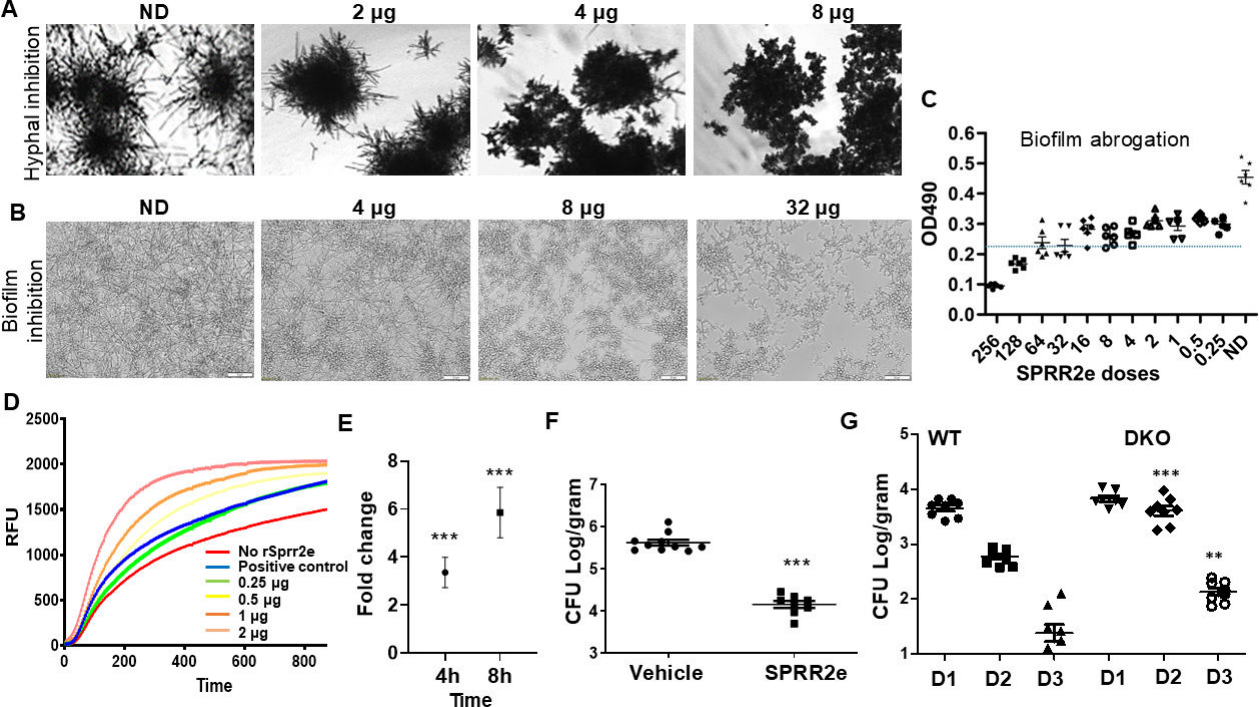
rSPRR2e has anti-*Candida* activity *in vitro* and *in vivo*. (**A**) rSPRR2e can prevent hyphal growth of *C. albicans* in RPMI incubated at 37°C, with the indicated doses for 12 h. (**B**) rSPRR2e, when incubated with 1 × 10^5^ cells/mL *C. albicans* yeast cells similarly in RPMI, can inhibit the filamentation and development of *C. albicans* biofilms at indicated doses. Pictures taken using brightfield microscopy at 40× or 10× magnification (**C**) rSPRR2e can disrupt preformed 24 h biofilms, measured by XTT assay at OD490 post-treatment for 24 h. Six replicates for each condition were included. All treated wells were significantly different (Mann-Whitney non-parametric, two-tailed test, *P* < 0.002) from the no drug control wells (ND); however, 50% inhibition was determined at 32 µg/mL (dotted line). (**D**) rSPRR2e can permeabilize *C. albicans* cell membranes measured by a fluorescent FDA assay. Positive control is *C. albicans* NDU1 mutant that has permeable membranes. The differences between no SPRR2e and SPRR2e doses >0.5 µg are statistically significant with *P* < 0.001. (**E**) SPRR2e is significantly expressed in TR146 oral keratinocytes during *C. albicans* infection versus uninfected cells. Results are an average of three replicates. (**F**) Treatment of 50 µg rSPRR2e intraorally for 3 days reduced *C. albicans* CFU in an OPC mouse model. Results are derived from an infection in 10 mice. (**G**) Sprr1a^−/−^ and Sprr2a^−/−^ knockout mice harbored significantly higher fungal CFU at days 2 and 3 of OPC. For statistical comparisons between the respective days in WT and double knockout mice (DKO), the Mann–Whitney U test (non-parametric, two-tailed) was applied to the fold change values. ***P < 0.0001, **P = 0.003.

We next evaluated the potency of SPRR proteins for their antifungal activity. For this, we recombinantly expressed tag-free *SPRR2e* proteins in yeast and performed MIC testing against *C. albicans*. We found that 0.5 µg/mL SPRR2e killed >80% of planktonically grown *C. albicans* cells (MIC_80_ = 0.5). SPRR2e could also prevent biofilm growth (performing MIC against a 100-fold higher cell number, 1 × 10^5^ cells, than the planktonic condition). A concentration of 4 µg/mL could inhibit hyphal proliferation (Fig. 7A), followed by abrogation of filamentation and biofilm growth at 8 µg/mL (Fig. 7B). Additionally, 32 µg/mL could destroy 50% of preformed mature biofilms (MIC_50_ = 32 µg/mL) (Fig. 7C).

SPRR proteins cause membrane permeability in bacteria (52). Hence, we questioned if they had a similar mode of action against *Candida*. Interestingly, we found that only 0.25 µg/mL of SPRR could disrupt membrane integrity and cause permeability to a level comparable to a *Candida* mutant gene (*ndu1/ndu1*) known to have compromised membrane integrity (54) (Fig. 7D).

The expression of SPRR was also measured *in vitro* in TR146 oral keratinocytes in the presence of *C. albicans*, which revealed a threefold and sixfold increase in SPRR levels post 4 and 8 h of infection, respectively (Fig. 7E). In mice with OPC, *Sprr* expression was the highest at day 2 of infection (>300-fold), and while its level subsequently tapered off, days 3 and 4 of OPC also displayed 30-fold and 7-fold higher expression than the uninfected tissue (Fig. S3B), indicating that *Sprr* expression is induced early and is sustained for a long period of time.

Furthermore, when rSPRR was used as intraoral treatment against fulminant OPC infection in immunocompromised mice, it provided >1.5 log reduction in CFU compared to the vehicle-treated control cohort (Fig. 7F). Given that SPRR proteins exhibited candidacidal activity *in vitro* and *in vivo*, we predicted that the removal of these proteins might promote OPC. To test this hypothesis, we used mice lacking SPRR1A and SPRR2A (*Sprr1a^−/−^*; *Sprr2a^−/−^*). Double knockout mice (DKO) and control mice infected with *C. albicans* had a significantly (1 log) higher fungal CFU than WT mice at day 2 (*P* < 0.0001). At day 2, when the wild-type mice had almost completely cleared the infection (limit of detection is Log 1), the DKO mice continued to significantly harbor 1 log higher CFUs (*P* < 0.003; Fig. 7G). Collectively, our results confirm that *SPRR* proteins are vital for resistance to OPC.

## DISCUSSION

Herein, we present an insight into the transcriptional landscape of the advanced stage of OPC. The studies performed in immunocompetent BALB/c mice served as a window to understand comprehensively the balance of immune response that helps clear the infection and initiate tissue repair. The UI tissue served as a baseline against which we examined changes in cell populations, their transcriptomes, and inferred interactions in the setting of OPC.

While BALB/c mice are often described as having a Th2-biased immune response, all mice strains are immunologically naïve to *C. albicans* (55), and adaptive immunity does not substantially contribute until at least 5–7 days post-infection. Our studies were conducted at day 2 post-infection in BALB/c mice, a time point dominated by the innate immune response. Besides, previous studies on OPC performed in ICR, C57BL/6 (Scott Fillers group) (56, 57), or BALB/c mice (Sarah Gaffen and Conti group) (30, 58) have demonstrated comparable outcomes in infection burden, fungal virulence, and early innate immune responses. These findings are further supported here, including the identification of oral megakaryocytes and the role of SPRR in antifungal defense, which appear conserved across mouse strains.

### Epithelial and stromal response

Our data highlight epithelial and stromal predominance at the infected tongue barrier, with processes like keratinization and angiogenesis altered by infection. Interestingly, these processes were altered differently in day 2-infected versus the uninfected tissues. While the majority of keratin genes (42 out of 54) were downregulated in the infected tissues, a handful of genes of the suprabasal keratinocytes considered as barrier alarmins were rapidly induced during wounding, including *Krt6a* and *Krt6b*, *Krt16* and *Krt17* (36) (Table S2). The *Krt6-Krt16* help withstand the rigors of an infected wound site, at the cost of a delay in epithelialization and optimal cell migration (36). Krt17 promotes Th-1/Th-17 profile for keratinocyte hyperproliferation (36). Remarkably, these genes, 3–10-fold up in infected tissues, were also highly active in the baseline unwounded/uninfected tongue epithelium, indicating a pre-primed repair state (Fig. S4B). Such priming is unique to oral buccal mucosa and not displayed by normal skin epithelial cells (34), which helps the oral mucosa repair at an accelerated pace compared to the skin (59). Interestingly, the epidermal differentiation complex genes, primed in buccal mucosa (60), were not pre-expressed in tongue, suggesting a distinct, infection-triggered immune response between the two oral sites.

Alongside downregulated keratins and collagen, ECM-degrading MMPs (*MMP3/8/9/13*) were upregulated, aiding immune cell recruitment. Tissue damage is known to trigger degradation of collagen in the ECM, which further enhances inflammation (61). Both degraded collagen and MMPs produced by activated monocytes/macrophages not only serve as a strong chemoattractant for immune cells but also promote migration of these cells (61). The infected tongue sections exhibited high strength of intercellular communication, indicative of cell-cell adhesion, growth factor production, and immunomodulation, indicative of early phases of hemostasis and inflammation in wound healing (Fig. 3A and B). Most outgoing signaling was from the stromal compartment, particularly the fibroblast to epithelial cells, influenced by the outermost cornified epithelial compartment that harbored *Candida*. The connection to hemostasis was further supported by the robust presence of over 40 genes involved in platelet activation, signaling, and degranulation in the infection milieu (Table S2). In addition to roles in blood clotting and wound healing, both megakaryocytes and the platelets these cells produce contribute to antifungal immune responses (62–65). In the oral mucosa, there is a tissue-localized population of megakaryocytes that responds to both IL-17 and candidalysin during OPC. Furthermore, C57Bl/6 mice genetically modified to lack megakaryocytes and platelets are highly susceptible to OPC compared to immunocompetent WT mice (30). Our finding of megakaryocytes also in the tongue tissue of Balb/c mice warrants future studies to better understand transcriptional regulation in these oral immune cells and the relevance to platelet production and activation during OPC in both strains of mice. Overall, the OPC environment represented an inflammatory phase for resistance to infection and wound healing.

### Inflammatory versus anti-inflammatory balance

One caveat of spatial transcriptomics is that each spot is occupied by more than one cell type, which does not provide data as unambiguous as single-cell transcriptomics. However, the technique has a major benefit that outweighs this disadvantage, i.e., the landscape of overlapping cell types and genes expressed can be visually pinpointed and quantified throughout the tissue as well as in the exact vicinity of infection, revealing the relative proportions of multiple cell types expressed simultaneously. Accordingly, the infected tissue displayed a threefold predominance in the breath of the anti-inflammatory immune response over the inflammatory genes in the keratinocytes of the infected tissue. While the type 2 IL-4 receptor IL-4Rα and IL-13Rα1 heterodimer were identified, the cytokine genes *IL-4* or *IL-13* that activate these receptors were not, perhaps indicating that the expression levels of these genes were too low or too early to detect. It has been reported that during the initiation phase of type 2 immune response, there is little or no type 2 cytokines present, and neutrophils are needed as a first wave of defense (66), including their process of degranulation and NET formation. Later, IL-4 and IL-13 suppress neutrophil effector functions, which would otherwise cause excessive tissue damage (67). Indeed, IL-4 and IL-13 signaling via the type 2 receptors orchestrate type 2 immunity to helminth infections (68) and can curtail chemotaxis and several effector functions of neutrophils in mice and humans, thereby mitigating detrimental tissue damage (66). In concordance with these reported findings, we found a high level of expression of a host of neutrophil degranulation/NETosis genes and pro-inflammatory cytokines (>60-fold) in the exact milieu of infection; yet there was also a larger breadth of presence of type 2 receptors in the same location at a comparatively lower ~3–4-fold average expression level (indicating the initiation phase of a type 2 response; Fig. 4B and E).

Based on these results, we expected the infection lesions to harbor both inflammatory M1 and anti-inflammatory M2 macrophages. In concurrence with the pro-inflammatory gene expression, M1 macrophages expressing *IL-1β, Tlr2,* and *Nos2* clustered around *Candida*, while despite higher expression of anti-inflammatory genes, M2 macrophages were largely omitted from within the infection niche and rather present around it. It is possible that the immediate infection environment focuses on getting rid of infection (via pro-inflammatory cytokines and M1), while simultaneously inducing factors for a balanced immune response and repair (anti-inflammatory genes). This dynamic milieu appeared to restrict the entry of M2 macrophages, which perhaps become accessible for their wound healing activity later, once the inflammation subsides and the fungus is eradicated.

### Infected versus resolved regions

Spatial transcriptomics allows for the simultaneous visualization and quantification of two different niches in the same tissue. INF regions used glycolysis, while RES zones relied on glycogenolysis and β-oxidation. These metabolic differences align with immune activity: glycolysis fuels rapid inflammation via platelet aggregation, HIF-1α induction, and cytokine expression (VCAM-1, CCL2), while β-oxidation supports immune resolution (46). Indeed, the infected tissues showed >5-fold overexpression of HIF-1α, a transcription factor that regulates glycolysis, increases the expression of activation markers such as *VCAM-1* (fourfold elevated), and pro-inflammatory chemokines such as CCL2 (40-fold upregulated) (69). INF regions showed enhanced neutrophil activity and metabolic pathways like fatty acid and cholesterol synthesis, crucial for antifungal signaling. Our studies revealed significant upregulation of metabolic pathways in inflamed epithelial cells, including fatty acyl-CoA, eicosanoid, sphingolipid, glycerophospholipid, and cholesterol biosynthesis. Eicosanoids like prostaglandins and leukotrienes enhance innate immune responses to *Candida* (70). Sphingolipids, cholesterol, and phospholipids contribute to lipid raft formation, key platforms for leukocyte signaling and migration (71). These findings suggest that immunometabolism shapes mucosal inflammation during *C. albicans* infection, offering potential therapeutic targets for inflammatory conditions like OPC and vulvovaginal candidiasis, especially in immunocompromised hosts.

In contrast, during the proliferative phase of wound healing, immune-regulatory cells such as Tregs and M2 macrophages rely on fatty acid oxidation via the TCA cycle for energy production (46, 47). Consistent with this, the infection-resolved tissue showed immune cell clearance and restoration of homeostasis, marked by increased collagen biosynthesis, mitochondrial fatty acid β-oxidation, and triglyceride catabolism. This region also displayed upregulation of genes involved in fibroblast activity, collagen and elastic fiber assembly, muscle contraction, and insulin-like growth factor (IGF) signaling. IGFs are known to modulate inflammation, support re-epithelialization (72), and regulate tissue development and metabolism (73, 74). Their potential role in resolving inflammation and promoting repair following mucosal candidiasis warrants further investigation.

### Pathways in cancer

*C. albicans* enhances the progression of oral squamous cell carcinoma (OSCC) and increases the incidence of esophageal cancer (4, 5, 75). Based on prior literature on the genetic mediators of early-onset tongue squamous cell carcinoma (TSCC) (76, 77) and on reports of OSCC factors triggered by *Candida* (75)*,* our study uncovered signatures of carcinogenesis during OPC. KEGG pathway analysis highlighted “pathways in cancer” as a major GO category, with upregulation of PI3K-Akt and MAPK pathway genes (e.g., *Egr1, Il-1β, Fosl, Fosl1, Fosl2, Junb*), TSCC-associated macrophage markers (Serpinb2, Osm), and myeloid-derived suppressor cell markers (Fcgrb3, Pfkfb3) were all >2-fold elevated. Nearly all cancer-related genes previously found to be altered in *Candida*-infected cancer cell lines, and several involved in TSCC, were also upregulated in our model (Fig. S5A). Some of the most highly expressed genes are localized to epithelial and endothelial cells within infected tissues (Fig. S5B).

These findings must be interpreted cautiously, as wound healing and tumorigenesis share overlapping molecular programs—leading to the idea that “a tumor is a wound that does not heal” (78). For instance, the *IL-6/JAK2/STAT3* axis (*Gp130, Ptgs2, Bcl2, Myc, Hbegf, Hif1α, Spp1*) was upregulated 6- to 30-fold during OPC, reflecting a dual role in tissue repair and oncogenesis (79). In immunocompetent hosts, this damage-repair response typically resolves post-infection. However, in immunocompromised individuals, sustained inflammation during OPC could potentially promote OSCC development. This underscores the importance of the anti-inflammatory axis, particularly M2 macrophages, which may either aid fungal persistence by dampening immunity or represent a host strategy to limit damage. Our findings support the latter: M2 macrophage-associated genes appeared by day 2 of infection, primarily in areas away from *Candida*, and were co-expressed with markers of wound healing, megakaryocyte differentiation, and platelet activation (80). Moreover, pharmacological depletion of M2 macrophages prolonged OPC beyond day 3, suggesting their essential role in infection resolution.

### SPRR mediates protection from OPC

SPRRs form a conserved multigene family clustered on chromosome 1q21 (81), comprising approximately eight SPRR2, two SPRR1, and one SPRR3 gene (82). These proteins are key components of the cornified envelope and were recently shown to possess antimicrobial activity through direct membrane disruption of bacteria (52). In OPC, the *SPRR1* and *SPRR2* genes were among the most highly upregulated (nearly 50-fold), suggesting a robust epithelial response. Given their roles in terminal keratinocyte differentiation, we questioned whether this induction stemmed from epithelial damage or a specific antifungal response to *Candida*.

As has been found in previous studies against helminth and bacterial infections (52, 83–85), recombinantly produced SPRR2e directly killed *C. albicans* yeast, hyphae, and mucosal biofilms at low microgram doses *in vitro* by disrupting plasma membrane permeability. *In vivo*, intraoral treatment with rSPRR2e significantly reduced fungal burden in the tongue. Moreover, *Sprr1a^–/–^Sprr2a^–/–^* DKO mice displayed higher fungal loads than wild-type controls, further validating the antifungal function of SPRR proteins.

SPRR expression appears to be regulated by innate cytokines. A recent study by Aggor et al. demonstrated that IL-17RA and IL-22RA signaling are essential for SPRR induction (86), and both receptors were upregulated in our data set. Converse to the SPRR expression by *C. albicans* that primarily infects the squamous epithelial layer of the tongue, penetration of bacterial stimuli to deeper portions of the skin has been implicated in SPRR production. MYD88 plays a role in SPRR protein production in skin, while interestingly, Lipopolysaccharide (LPS) is not able to stimulate its production in mouse keratinocytes (52). Future studies could examine the role of candidalysin in triggering SPRR, considering that the toxin induces alarmins and other AMP in epithelial cells possessing potent anti-*Candida* functions (87). Proline-rich AMP sourced from other systems (plants, insects) can modulate the immune system via cytokine activity or angiogenesis and penetrate microbial cell membranes (88). Ongoing studies of these peptides carry the potential to serve as important adjuncts to existing antifungal drug treatment.

### Conclusion

The present study utilized spatial genomics to provide a visual landscape of the host response to OPC beyond the pro-inflammatory immune reaction that has been exhaustively studied. These findings add significant insights into the juxtaposition and expression of both the anti-inflammatory and wound repair genes, the important role of M2 macrophages, and the antifungal potential of a new family of antimicrobial proteins. Limitations of this study are the absence of a temporal analysis of early time points of infection and the restricted mechanistic evaluation of identified genetic correlates. Nevertheless, this is one of the first studies that delves into the anti-inflammatory realm of mucosal candidiasis that persuades a balance of immunity for eradicating OPC in an immunocompetent background.

## MATERIALS AND METHODS

### Strain and culture conditions

The *C. albicans* strain used in this study is SC5314 (89). Stock cultures of all strains were stored in 15% glycerol at −80°C. Strains were routinely grown under yeast conditions (media at 30°C) in YPG (1% yeast extract, 2% Bacto peptone, 2% glucose) or under filament-inducing conditions using RPMI medium (Sigma, St. Louis, MO) with MOPS (morpholinepropanesulfonic acid) buffer or Spider medium.

### Mouse model of oropharyngeal candidiasis

Male 6-week-old Balb/c mice were purchased from Taconic. OPC was induced in mice as described previously (90). For the spatial transcriptomics study, immunocompetent mice were used to understand the comprehensive immune response to infection. For infection, the animals were sedated, and a swab saturated with 2 × 10^7^ *C. albicans* cells was placed sublingually for 75 min. Mice were killed at different time points post-infection. The tongues were harvested, weighed, homogenized for 30 s, and quantitatively cultured to enumerate CFUs. For histopathology, some tongues were fixed in zinc-buffered formalin, embedded in paraffin, sectioned, and stained with PAS. For spatial transcriptomics studies, the tongue tissues were rapidly harvested and flash frozen in liquid nitrogen. Frozen tongues were stored at −80°C. Tongues were embedded in optimal cutting temperature medium and cryosectioned at −20°C (10 μm sections) using CryoStar NX50 (Kalamazoo, MI) at the Department of Pathology, UCLA.

For measuring the efficacy of recombinant SPRR against OPC, mice were treated intraorally, by delivering 50 μg of recSPRR2e (Cusabio, Houston, TX) in 25 μL using a pipette tip on days 1 to 3. Mice were killed on day 4 after infection. The tongues were harvested, weighed, homogenized for 30 s, and quantitatively cultured. Similarly, for examining the importance of M2 macrophages in optimal clearance of OPC, mice were treated IP with 125 μg of the M2 macrophage inhibitor Rp832 on days −1 and +1 following infection. Tongues of drug-treated and untreated mice were harvested at day 2 (D2) and D3 post-infection, homogenized, and plated for fungal CFUs that were plotted in GraphPad Prism 5.

For understanding the role of endogenously produced SPRR proteins on resistance to infection, OPC was developed in C57BL6 *Sprr1a*^−/−^; *Sprr2a*^−/−^ or WT male mice as described above, and the fungal burden in the tongues was measured by CFU measurement. Sprr1a^−/−^; Sprr2a^−/−^ mice were generated using CRISPR/Cas9 (52) by the Harris-Tryon group and generously provided to us for our studies.

### Tissue processing and Visium data generation

Tissue section was mounted on Visium Spatial Gene Expression Slides (catalog no. 2000233, 10× Genomics) and stained with H&E. Brightfield images of the H&E-stained sections were acquired (20×) and stitched together using the Leica Aperio AT2 scanner. Tissue optimization was performed to determine the optimal permeabilization time for tongue tissue for the downstream gene expression protocol. Spatial transcriptomics was performed on tongue cryosections using the Visium Spatial Gene Expression Slide & Reagent Kit, 16 reactions (Catalog # PN-1000184), according to the manufacturer’s protocol (10× Genomics, Pleasanton, CA, USA). Briefly, sections were enzymatically permeabilized for 12 min. Permeabilization resulted in the release of polyA mRNA from the tissue, enabling capture by poly(dT) primers precoated on the Visium Gene Expression slides. Slides also contained barcoded probes with unique molecular identifiers (UMI) so that the spatial gene distribution was mapped. After reverse transcription and second-strand synthesis, the amplified cDNA samples from the Visium slides were transferred, purified, and quantified for library preparation. cDNA quantification was performed using the Agilent Bioanalyzer High Sensitivity Kit on an Agilent 4200 TapeStation System (Agilent, G2991BA). cDNA libraries were sequenced on an Illumina NovaSeq 6000 sequencer for PE 2X100 run using the SP flowcell (100 cycles) at the UCLA Technology Center for Genomics & Bioinformatics (TCGB). A data quality check was done on Illumina SAV. Demultiplexing was performed with Illumina Bcl2fastq v2.19.1.403 software.

Fastq files were mapped to the reference genome mm10-2020-A, and UMIs were quantified using 10× Space Ranger (v2.0). Barcodes from each sample were selected using the drawing tool in Loupe Browser and then exported to the Seurat package (v5.0.1) (91). Samples were integrated using SpaceRanger aggr. Normalization and variance stabilization of molecular count data were done with the R package Seurat (v4) to improve downstream analytical tasks, including gene selection, dimensional reduction, and differential expression from spatial data sets. Capture spots were normalized using SCTransform (92) utilizing the default parameters, followed by the standard Seurat dimensionality reduction and clustering workflow using the top 30 components. The FindAllMarkers function was used to identify the top marker genes of each cluster, and capture spots were annotated manually using canonical markers for each expected cell type in conjunction with the marker genes of each cluster. Seurat objects of each sample were merged with the merge function, followed by a joint dimensional reduction and clustering analysis using the top 30 components. The SpatialFeaturePlot function was used to visualize gene expression of individual genes across the tissue section. The average gene expression of selected genes was visualized in each group and each cluster using the Seurat DotPlot and VinPlot functions. Gene expression heatmaps were generated using Python (v3.10) with the Seaborn and Matplotlib libraries. Log₂ fold changes (OPC vs UI) were plotted. Genes were grouped by functional categories, and annotated sidebars were used to indicate AMP, cytokine, chemokine, receptor, remodeling, or signaling functions. Heatmaps were rendered using clustermap with bwr or RdYlGn_r color palettes.

### Quantitative RT-PCR

The frozen tongue tissues (four independent biological replicates) were ground in liquid nitrogen as previously described by us (93), and RNA extracted using the RNeasy kit (Qiagen, Germantown, MD). The RNA preparation was DNAse-treated, and the absence of DNA contamination was confirmed with the housekeeping gene GAPDH. RNA quality and quantity were determined (93), cDNA was synthesized from known amounts of total RNA, and equal amounts of cDNA were used as starting templates for RT-PCR reactions. Analysis of the transcript was carried out using SYBR Green PCR Master Mix (Applied Biosystems, Foster City, CA) in an ABI Prism 7300 Sequence Detection System (Applied Biosystems). Each reaction was set up in triplicate in a 25.0 μL volume with 1.0 μL cDNA for 40 cycles (thermal cycling conditions: initial steps of 50°C for 2 min and 95°C for 10 min; then 40 cycles of 95°C for 15 s; 60°C for 1 min). The target genes were normalized to GAPDH. Relative gene expression was quantified by the 2–ΔΔCT method and analyzed using the inbuilt ABI analysis software.

### SPRR2e measurement in TR146 cells

*In vitro* experiments were carried out using the TR146 human buccal epithelial squamous cell carcinoma cell line (94), obtained from the European Collection of Authenticated Cell Cultures and cultured in Dulbecco’s Modified Eagle’s Medium (DMEM, Sigma-Aldrich), supplemented with 10% fetal bovine serum (FBS) and 1% penicillin-streptomycin. Serum-free DMEM was used to replace normal growth medium 24 h before and during the experimentation process. TR146 cells were infected with 1 × 10^5^ cells/mL *C. albicans* and incubated for 4 and 8 h at 37°C, at which time the wells were washed twice with ice-cold PBS and flash frozen using liquid nitrogen. RNA was extracted from the frozen cells and subjected to qRT-PCR (as described above) for measurement of SPRR gene expression.

### Biofilm growth assay

*C. albicans* biofilms were formed in 24-well microtiter plates as previously described (95). Briefly, 1 mL of *C. albicans* cells (1 × 10^6^ cells/mL) is added to the wells of a 24-well microtiter plate and incubated overnight in RPMI, and the biofilms are gently washed two times. These biofilms were quantified using the XTT assay (95) and read spectrophotometrically at OD490. To evaluate the effect of SPRR on filamentation and biofilm growth, the recombinantly produced SPRR2e was added at different concentrations (0 to 32 μg) to the wells at the time of biofilm initiation. The fungal cells were allowed to interact with the recombinant protein for 20 h, after which the morphology and biofilm growth were quantified, microscopically or by XTT assay, respectively. Recombinant SPRR2e was also added to an overnight pre-formed *C. albicans* biofilm, and the inhibitory effect of the protein was monitored after another 24 h and measured as above. An MIC for SPRR was also performed against *C. albicans* planktonic and biofilm cells, using CLSI standards (CLSI M27-A3) and as previously described (96, 97).

### Cell membrane permeability

*C. albicans* was grown in YPG for 48 h, and about 5 × 10^6^ cells were resuspended and washed twice in 1 mL of fluorescein diacetate (FDA) buffer before supplementing with 50 nm FDA. A 200-μL volume of cell mixture with or without FDA, and in the presence and absence of different concentrations of recombinant SPRR protein, was added to an optical-bottom 96-well plate. *C. albicans* NDU1 mutant was used as a positive control displaying maximum cell membrane permeability (98). The kinetics of FDA uptake were recorded every 5 min for 30 reads with simultaneous shaking of samples in a plate reader with excitation and emission wavelengths of 485 and 535 nm, respectively. Data represent the fluorescence intensity over time.

### Flow cytometry

For oral immune cell analysis, mice were sacrificed, and tongue tissue harvested, then mechanically homogenized in RPMI 1640 media and incubated at 37°C for 45 min on a GentleMACS Dissociator (Miltenyi Biotec) using the Murine Tissue Dissociation Kit, then passed through a 40 mm cell strainer to form single-cell suspensions. After brief centrifugation, cells were reconstituted with PBS supplemented with 2% FBS and 2 mM EDTA. 1 × 10^6^ cells/mL were obtained by staining with Trypan blue and counting on a hemocytometer. An initial incubation of CD16/CD32 Fc Block (BD Biosciences) was followed by staining with the following antibodies, all from BioLegend: CD45-PE/Cy7, CD11b-PE/Cy7, GR-1-BV510, CD41-BV421, CD62p-APC, CD42d-PerCP/Cy5, and F4/80-PE. All flow cytometry was performed on an LSRFortessa (BD Biosciences). To detect megakaryocytes in murine tongue tissue, doublets were first removed using a gate on the FSC-A versus FSC-H plot, followed by a general live cell gate to remove cellular debris. Within the live cell gate, megakaryocytes were defined as positive for CD41 and negative for CD11b. Neutrophils were classified as GR-1- and CD11b-positive. Analyses of flow cytometry results were performed using FlowJo (BD Bioscience).

## SUPPLEMENTAL MATERIAL

Supplemental figures mbio.00849-25-s0001.pdf Figures S1 to S5.

Legends mbio.00849-25-s0002.docx Legends for the supplemental figures.

Table S1 mbio.00849-25-s0003.xlsx Annotations of cell type markers.

Table S2 mbio.00849-25-s0004.xlsx Significantly regulated host and *Candida* genes including the top GO terms.

Table S3 mbio.00849-25-s0005.csv Differentiation of macrophage cell types.

Table S4 mbio.00849-25-s0006.xlsx Features of M1 and M2 macrophage genes.

Table S5 mbio.00849-25-s0007.xlsx Significant differential regulation of host ECM and wound healing genes.

Table S6 mbio.00849-25-s0008.csv IL-24 features.

Table S7 mbio.00849-25-s0009.xlsx Gene expression differences between the resolved versus infected tongue.

Table S8 mbio.00849-25-s0010.csv SPRR2e features.

ASM does not own the copyrights to Supplemental Material that may be linked to, or accessed through, an article. The authors have granted ASM a non-exclusive, world-wide license to publish the Supplemental Material files. Please contact the corresponding author directly for reuse.
