# Supplementary material for: A spatial transcriptomic atlas of the host response to oropharyngeal candidiasis
Source: mBio. 2025 Jun 30;16(8):e00849-25. doi: 10.1128/mbio.00849-25 (PMC12345175; doi:10.1128/mbio.00849-25)
Supplement: Supplemental figures — Figures S1 to S5. [file mbio.00849-25-s0001.pdf]

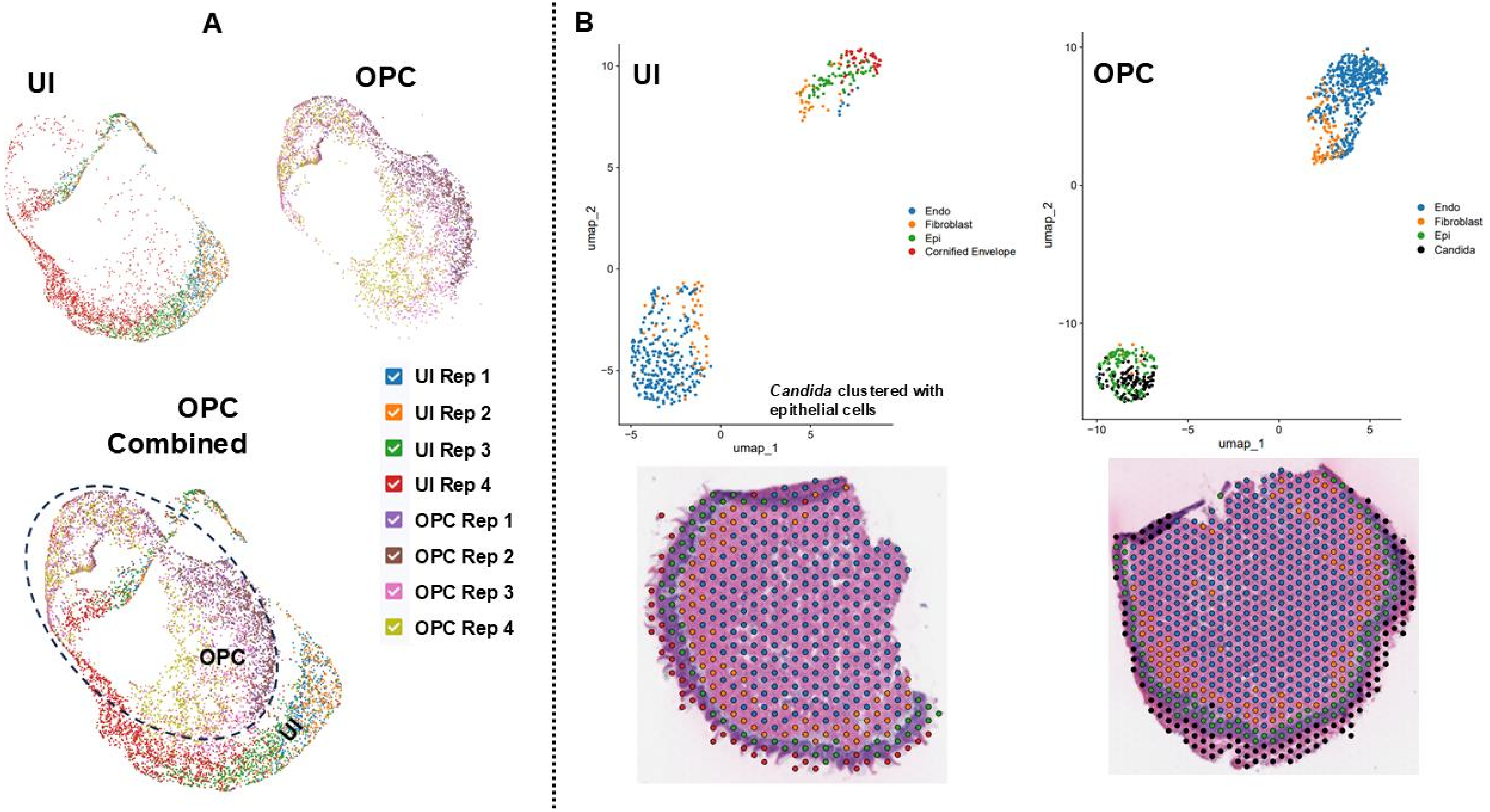
A
UI
OPC
OPC Combined
OPC
UI
UI Rep 1
UI Rep 2
UI Rep 3
UI Rep 4
OPC Rep 1
OPC Rep 2
OPC Rep 3
OPC Rep 4
B
UI
umap_2
umap_1
Endo
Fibroblast
Epi
Cornified Envelope
Candida clustered with epithelial cells
OPC
Endo
Fibroblast
Epi
Candida

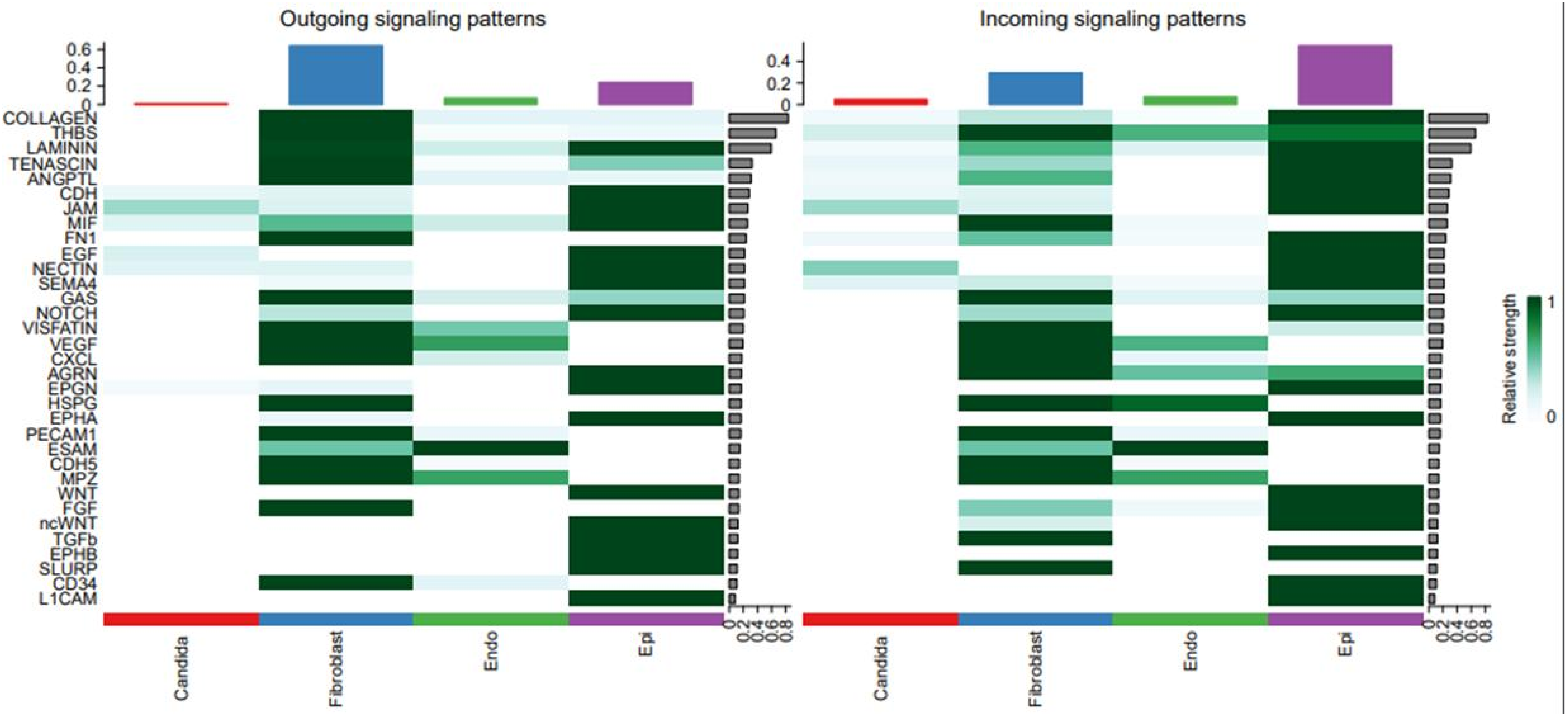

Outgoing signaling patterns
Incoming signaling patterns
COLLAGEN
THBS
LAMININ
TENASCIN
ANGPTL
CDH
JAM
MIF
FN1
EGF
NECTIN
SEMA4
GAS
NOTCH
VISFATIN
VEGF
CXCL
AGRN
EPGN
HSPG
EPHA
PECAM1
ESAM
CDH5
MPZ
WNT
FGF
ncWNT
TGFb
EPHB
SLURP
CD34
L1CAM
Candida
Fibroblast
Endo
Epi
Relative strength

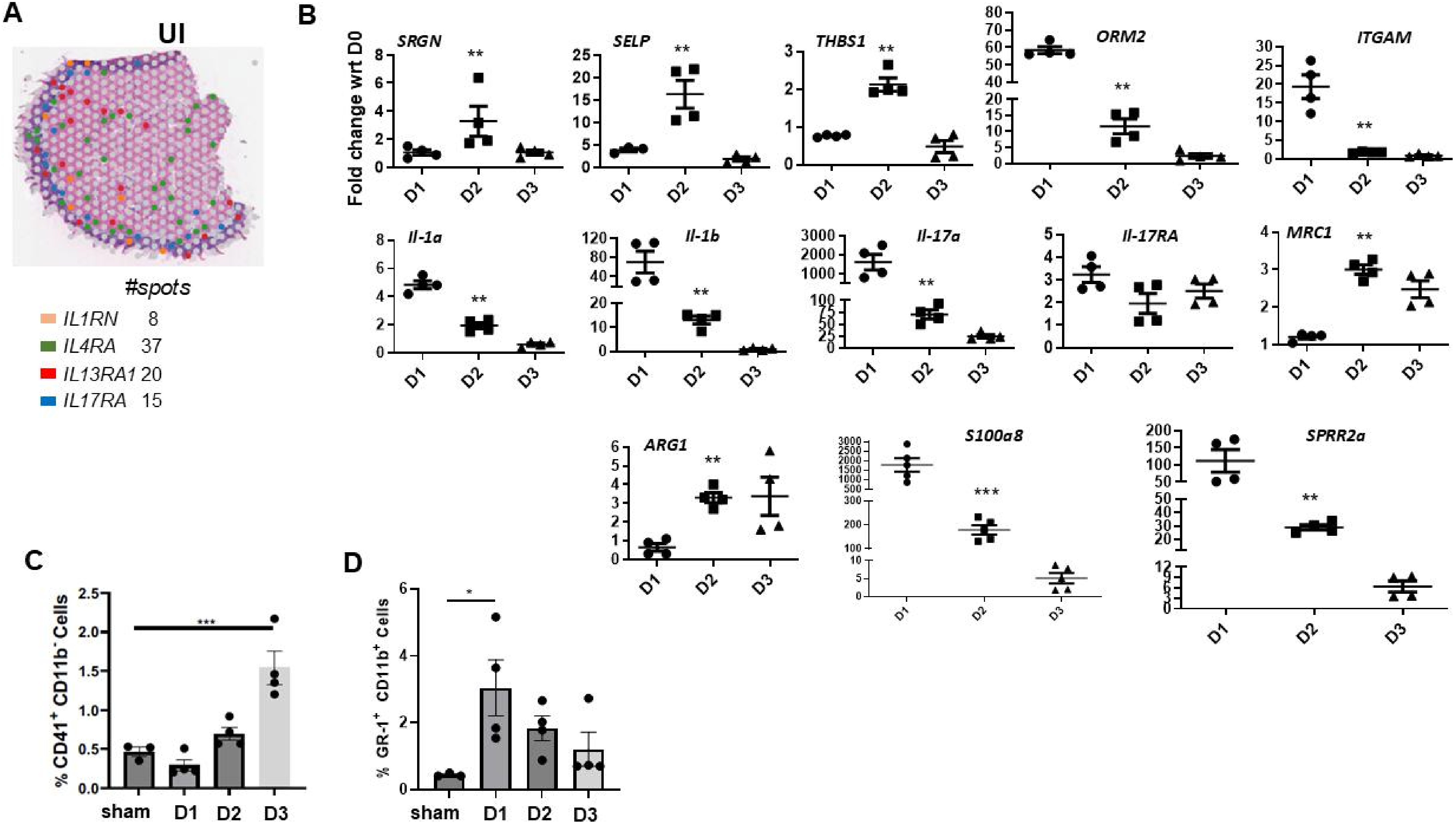

A
UI
#spots
IL1RN 8
IL4RA 37
IL13RA1 20
IL17RA 15
B
Fold change wrt D0
SRGN
SELP
THBS1
ORM2
ITGAM
Il-1a
Il-1b
Il-17a
Il-17RA
MRC1
ARG1
S100a8
SPRR2a
D1
D2
D3
C
% CD41+ CD11b- Cells
sham
D1
D2
D3
D
% GR-1+ CD11b+ Cells
sham
D1
D2
D3

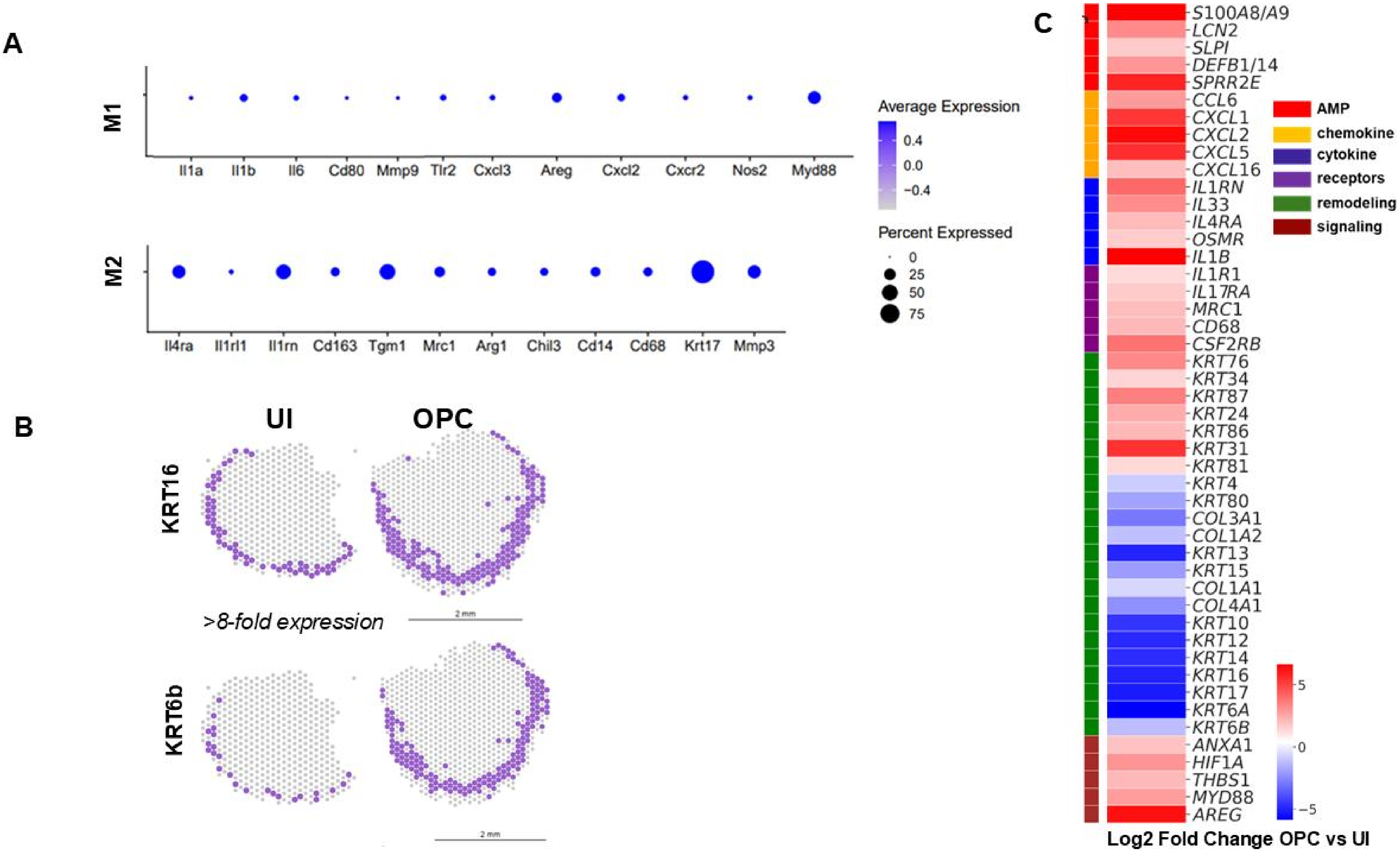

A
M1
Il1a
Il1b
Il6
Cd80
Mmp9
Tlr2
Cxcl3
Areg
Cxcl2
Cxcr2
Nos2
Myd88
M2
Il4ra
Il1rl1
Il1rn
Cd163
Tgm1
Mrc1
Arg1
Chil3
Cd14
Cd68
Krt17
Mmp3
Average Expression
0.4
0.0
−0.4
Percent Expressed
0
25
50
75
B
UI
OPC
KRT16
>8-fold expression
2 mm
KRT6b
2 mm
C
S100A8/A9
LCN2
SLPI
DEFB1/14
SPRR2E
CCL6
CXCL1
CXCL2
CXCL5
CXCL16
IL1RN
IL33
IL4RA
OSMR
IL1B
IL1R1
IL17RA
MRC1
CD68
CSF2RB
KRT76
KRT34
KRT87
KRT24
KRT86
KRT31
KRT81
KRT4
KRT80
COL3A1
COL1A2
KRT13
KRT15
COL1A1
COL4A1
KRT10
KRT12
KRT14
KRT16
KRT17
KRT6A
KRT6B
ANXA1
HIF1A
THBS1
MYD88
AREG
AMP
chemokine
cytokine
receptors
remodeling
signaling
5
0
−5
Log2 Fold Change OPC vs UI

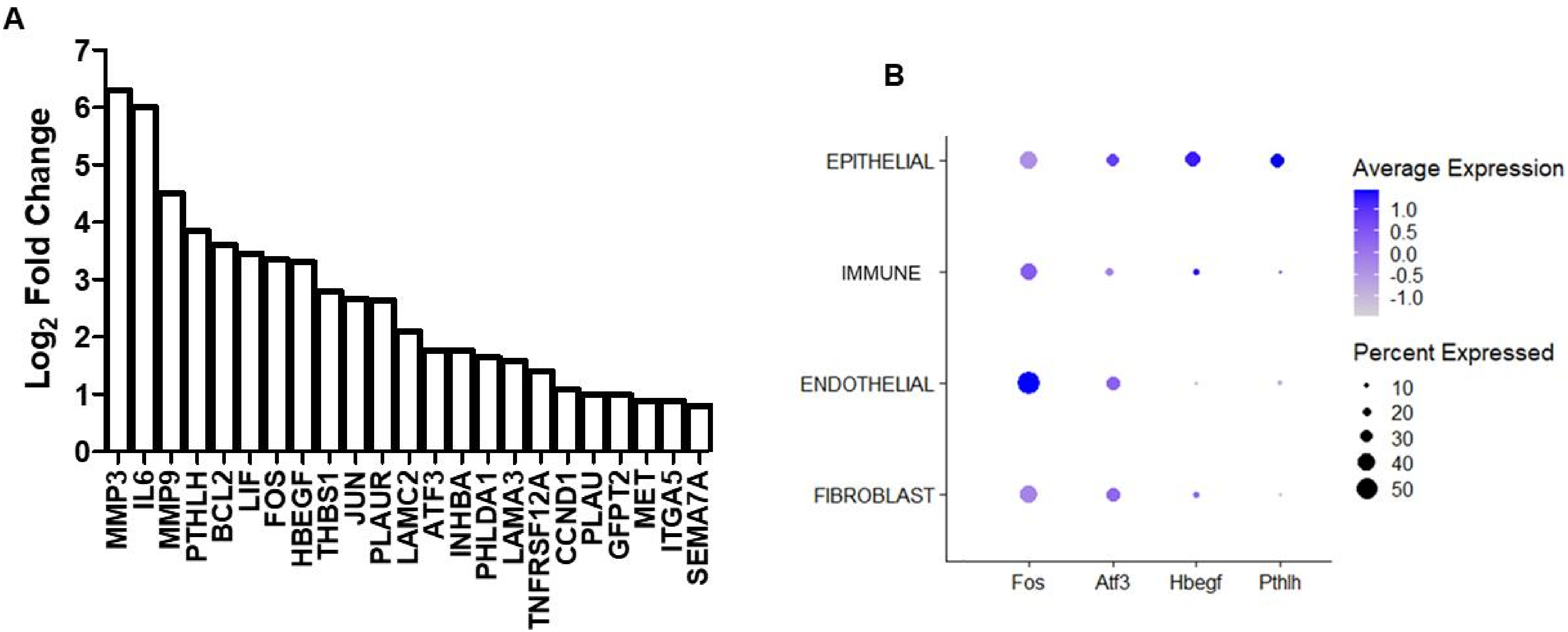
A
Log2 Fold Change
7
6
5
4
3
2
1
0
MMP3
IL6
MMP9
PTHLH
BCL2
LIF
FOS
HBEGF
THBS1
JUN
PLAUR
LAMC2
ATF3
INHBA
PHLDA1
LAMA3
TNFRSF12A
CCND1
PLAU
GFPT2
MET
ITGA5
SEMA7A
B
EPITHELIAL
IMMUNE
ENDOTHELIAL
FIBROBLAST
Fos
Atf3
Hbegf
Pthlh
Average Expression
1.0
0.5
0.0
-0.5
-1.0
Percent Expressed
10
20
30
40
50
