# Supplementary material for: A spatial transcriptomic atlas of the host response to oropharyngeal candidiasis
Source: mBio. 2025 Jun 30;16(8):e00849-25. doi: 10.1128/mbio.00849-25 (PMC12345175; doi:10.1128/mbio.00849-25)
Supplement: Legends — for the supplemental figures. [file mbio.00849-25-s0002.docx]

**Supplemental data Legends**

**Figure S1. A. PCA of replicates.** Four UI and four D3 replicates (two biological and two technical replicates each condition) were utilized for this study. PCA analyses revealed a statistically significant co-relation between the individual group replicates and enough separation between the two groups. **B. Cell type annotations.** UMAP representation (upper panel) of cell types identified on OPC vs UI. Note the cornified layer is overrun by C. albicans in OPC tissues. Bottom panel visually depicts the spatially resolved cell types.

**Figure S2. Cell chat identified signaling direction and genes.** Additional genes communicating between cell types

**Figure S3. qRT-PCR verification of genes differentially expressed in the spatial study. (A)** UI tissues had a very low baseline level of expression of type 1 and type 2 genes. (**B**) A time-course qRT-PCR verification of DEG data (4 independent replicates). For statistical comparisons between D1 and D2, the Mann–Whitney U test (non-parametric, two tailed test) was applied to the fold change values. Statistical significance was denoted as: ***p < 0.01, **p < 0.03 (**C**) Sorting of MK cells from Balb/c mice at different timepoints to measure their content during OPC. ***p<0.01. (**D**) Sorting of neutrophil numbers at different timepoints during OPC. *P<0.01.

**Figure S4. Abundance and importance of M2 macrophages. (A)** Dot plots showing increased expression and numbers of M2 macrophages versus M1 in OPC (**B**) Spatial representation of baseline expression levels of keratin genes in UI. (**C**) Heat map displaying Log₂ fold changes (OPC vs UI). Genes are grouped by functional categories and indicated by the annotated sidebar.

**Figure S5. Expression of oral cancer-associated genes in OPC versus UI. (A)** Graph showing log2 fold change of OPC-induced genes in mice tongues. (**B**) Dot plot of the expression patterns and abundance of four oral cancer-associated genes and four different cell types
